# The Performance Index Identifies Changes Across the Dual Task Timed Up and Go Test Phases and Impacts Task-Cost Estimation in the Oldest-Old

**DOI:** 10.3389/fnhum.2021.720719

**Published:** 2021-09-30

**Authors:** Fabiane Oliveira Brauner, Gustavo Balbinot, Anelise Ineu Figueiredo, Daiane Oliveira Hausen, Aniuska Schiavo, Régis Gemerasca Mestriner

**Affiliations:** ^1^Graduate Program in Biomedical Gerontology, School of Medicine, Pontifical Catholic University of Rio Grande do Sul, PUCRS, Porto Alegre, Brazil; ^2^Neuroplasticity and Neural Repair Research Group, Health and Life Sciences School, Pontifical Catholic University of Rio Grande do Sul, PUCRS, Porto Alegre, Brazil; ^3^KITE - Toronto Rehabilitation Institute, University Health Network, Toronto, ON, Canada

**Keywords:** dual task, oldest-old adults, falls, timed up and go test, gait, cognition

## Abstract

**Introduction:** Dual tasking is common in activities of daily living (ADLs) and the ability to perform them usually declines with age. While cognitive aspects influence dual task (DT) performance, most DT-cost (DT-C) related metrics include only time- or speed- delta without weighting the accuracy of cognitive replies involved in the task.

**Objectives:** The primary study goal was to weight the accuracy of cognitive replies as a contributing factor when estimating DT-C using a new index of DT-C that considers the accuracy of cognitive replies (P-index) in the instrumented timed up and go test (iTUG). Secondarily, to correlate the novel P-index with domains of the Mini-Mental State Examination (MMSE).

**Methods:** Sixty-three participants (≥85 years old) took part in this study. The single task (ST) and DT iTUG tests were performed in a semi-random order. Both the time taken to complete the task measured utilizing an inertial measurement unit (IMU), and the accuracy of the cognitive replies were used to create the novel P-index. Clinical and sociodemographic data were collected.

**Results:** The accuracy of the cognitive replies changed across the iTUG phases, particularly between the walk 1 and walk 2 phases. Moreover, weighting 0.6 for delta-time (W_1_) and 0.4 for cognitive replies (W_2_) into the P-index enhanced the prediction of the MMSE score. The novel P-index was able to explain 37% of the scores obtained by the fallers in the “spatial orientation” and “attention” domains of the MMSE. The ability of the P-index to predict MMSE scores was not significantly influenced by age, schooling, and number of medicines in use. The Bland-Altman analysis indicated a substantial difference between the time-delta-based DT-C and P-index methods, which was within the limits of agreement.

**Conclusions:** The P-index incorporates the accuracy of cognitive replies when calculating the DT-C and better reflects the variance of the MMSE in comparison with the traditional time- or speed-delta approaches, thus providing an improved method to estimate the DT-C.

## Introduction

Fall-related injuries are a public health problem worldwide (Sherrington et al., 2017) and falling while performing activities of daily living (ADLs) is common in older adults (Tomas-Carus et al., 2019). Bone fractures, hospitalization, depression, and sedentary behavior are among the fall-triggered health issues that contribute to reducing the quality of life and autonomy (Ambrose et al., 2013). Understanding the interaction between fall-related factors is important to detect and prevent falls in older adults, which is of utmost clinical importance.

ADLs require different levels of cognitive demands and may involve dual tasks (DTs; e.g., walking in the supermarket while remembering what goods should be bought) (Herold et al., 2018). Commonly, the DT approach is used in laboratory settings to assess the ability to deal with DTs during simulated ADLs. DT approaches often utilize a cognitive factor to interfere with motor performance. In such cognitive-motor DT, the between-systems interplay may reduce the performance in the task, and the interference level depends on DT type, complexity, and individual skills (Bayot et al., 2018; Yiu et al., 2018). Cognitive-motor DTs, may require good levels of executive function, working memory, as well as attention, language, among other neurocognitive functions while performing a simultaneous motor gesture (Ehsani et al., 2019; Rhodes et al., 2019). For instance, attention is needed to perceive the environmental context and may be shared, alternated, or concentrated in one or more targets (Shalev et al., 2016). Thus, attention constraints may influence the cognitive load by increasing the working memory demands. In some cases, it hampers the ability to retain, retrieve, and recall information in a short period (Rodrigues and Pandeirada, 2015). By contrast, an optimal executive function may mitigate DT interference during walking (Bridenbaugh and Kressig, 2015). These are examples of how the cognitive load may affect multi-task performance, which would be reflected in the time taken to complete the task [i.e., conventional DT-cost (DT-C) metrics].

Older adults frequently experience both neurocognitive and neuromuscular decline, which contributes to reducing performance in walking-based DTs, with respectively, greater risk of falls (Rosso et al., 2018). Hence, we can assume an increased risk of falls within increased age in older adults (Kafri et al., 2017). Several studies have addressed functional mobility in older adults using different DT interferences (Yogev-Seligmann et al., 2012; Patel et al., 2014; Brustio et al., 2018; Commandeur et al., 2018) and most of these studies employed the instrumented timed up and go test (iTUG) to assess functional mobility (Zaferiou et al., 2017; Howcroft et al., 2018; Patel et al., 2019; Figueiredo et al., 2020).

Despite considerable efforts, identifying the best DT to assess the risk of falls in the oldest-old (individuals aged ≥ 80 years) remains a challenge (Montero-Odasso et al., 2019) and several types of cognitive-motor tasks have been investigated in fall-risk assessment trials (Barbosa et al., 2008; Fatori et al., 2015; Yeo, 2017; Asai et al., 2018; Zirek et al., 2018; Gillain et al., 2019; Allahverdipour et al., 2020; Figueiredo et al., 2020). For example, repeating the weekdays in reverse order during a cognitive-motor DT requires dividing attention and temporal organization to select the correct days while walking (Yeo, 2017). All these cognitive-motor tasks may lead to increased time- or speed- delta between single task (ST) and DT, captured by the traditional equations to estimate the DT-C (Kelly et al., 2010; Asai et al., 2018; Hunter et al., 2018).

Selecting the most appropriate equation to estimate the DT-C is challenging (Venema et al., 2019). Usually, the calculation of the DT-C considers the time- or speed-delta between the ST and DT (Kelly et al., 2010; Asai et al., 2018; Hunter et al., 2018). Nonetheless, most of the equations disregard the accuracy of the cognitive replies, which may influence time or speed differences in the test (Bherer et al., 2008; Tomas-Carus et al., 2019). The accuracy of cognitive replies can be obtained by counting the number of total, correct and wrong replies during the DT. The incorporation of the accuracy of cognitive replies into the DT-C calculation (P-index) may enhance the association of the DT assessment with neurocognitive decline, and better reflect the engagement and performance during the task. Primarily, the present study sought to weight the accuracy of cognitive replies as a contributing factor to estimate DT-C in the iTUG test using a novel modified P-index. We also tested the association of the P-index with neurocognitive performance by exploring correlations between the weighted P-index and mini-mental state examination (MMSE) cognitive domains. Secondarily, we compared the traditional time- or speed- delta DT-C and P-index in the oldest-old with and without a history of falls.

## Materials and Methods

### Participants

This is an observational, cross-sectional study. Participants were recruited (fallers and non-fallers) by convenience in the city of Porto Alegre, Rio Grande do Sul–Brazil. After an initial telephone call, a home visit was scheduled (lasting approximately 1 h and 30 min) to collect the data. The inclusion criteria were: volunteers of any gender; aged ≥ 85 years, who were able to walk independently (walking-assistant devices allowed), and understood the verbal commands necessary to complete the assessment test battery. The exclusion criteria were: participant uncertainty regarding their history of falls in the 6 months prior to the assessment (information was cross-checked with relatives or caregivers); hospitalization for more than 7 days in the previous 3 months; and a diagnosis of neurological (including major cognitive decline or dementia), severe respiratory, cardiovascular, visual, or auditory diseases (self-reported and confirmed by medical contact or report). Clinical and sociodemographic information (age, gender, blood pressure, schooling, marital status, medications in use, ethnicity, smoking, drinking, and history of falls) were collected during the assessment interview. This research was approved by the local Research Ethics Committee (number 2.278.707). All volunteers signed a free and informed consent form.

### Falls

The volunteers who self-reported having fallen in the 6 months prior to the assessment were classified as “fallers” and those who did not report this experience were considered “non-fallers” (Hughes et al., 2020). A fall was defined as an involuntary and unexpected event in which the participant comes to rest on the ground (Deandrea et al., 2010). This definition was used because minor falls, i.e., unintentional changes in body level, without impacting on the ground, are more likely to be forgotten.

### Instrumented Timed Up and Go Test (iTUG)

**FIGURE 1:**
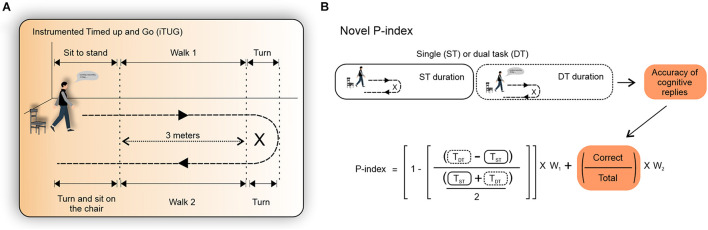
The instrumented timed up and go (iTUG) test, the dual task cost (DT-C), and the novel performance index (P-index). **(A)** The iTUG test. **(B)** The accuracy of the cognitive replies during the DT (orange–defined as the ratio of correct vocalizations and total vocalizations) was included in the time-delta equation for calculating the cost of the dual task (DT-C) (Asai et al., 2018). The P-index is a novel index adjusted by the accuracy of the DT. Higher P-index values indicate greater DT performance. We tested the influence of the cognitive task (i.e., correct/total vocalizations) by increasing W_2_ and decreasing W_1_ at 0, 0.1, 0.2, 0.3, 0.4, 0.5, 0.6, 0.7, 0.8, 0.9, and 1, respectively.

An inertial measurement unit (IMU) with a sampling rate of 100 Hz was used during the ST and DT (Bluetooth-compatible IMU, G-Walk, BTS Bioengineering, MA, United States). The IMU was positioned between the L5 and S1 vertebrae using an elastic belt provided by the manufacturer (Kleiner et al., 2017; Pau et al., 2018). Two types of iTUG tests were performed in a semi-random order (ST—DT—DT—ST—DT). We used three trials of each iTUG type to obtain a representative performance while minimizing fatigue in the oldest-old volunteers (Witchel et al., 2018; Figueiredo et al., 2020). Participants were asked to walk as fast as possible during the iTUG tests. To begin, the participants were seated on a standard chair provided by the researcher (43 cm in height, without armrests). The verbal command “get up and go” was given and participants were expected to walk for 6 m (3 m in the forward gait phase—walk 1, perform a 180° turn—turn phase, and return 3 m—walk 2 phase, and sit in the same chair from which they had started—turn and sit phase) (Figure 1A). During the DT iTUG, participants followed the same above-mentioned protocol while vocalizing the weekdays in reverse order (e.g., Wednesday, Tuesday, Monday, and so on until the completion of the test) (Barbosa et al., 2008; Fatori et al., 2015; Allahverdipour et al., 2020; Figueiredo et al., 2020). Participants were instructed to start speaking, as quickly as possible, while still sitting. At each DT iTUG trial, the initial day of the week was changed to reduce the chances of automatizing the sequence (Figueiredo et al., 2020). Each volunteer was previously informed that the correct/wrong answers would be recorded.

### Cognition

The MMSE was used as a screening test for cognitive decline (Folstein et al., 1975; Bertolucci et al., 1994). The MMSE was developed by Folstein et al. (1975) and was adapted to Brazilian Portuguese by Bertolucci et al. (1994). The total MMSE score and the temporal orientation, spatial orientation, attention, memory, and language domains were assessed (Bertolucci et al., 1994; Kochhann and Lisboa, 2010).

### Weighting the Accuracy of Cognitive Replies in the DT iTUG Performance

The influence of weighting cognitive replies to estimate DT-C was tested using an adapted version of the traditional time-delta equation (Asai et al., 2018) (Eq. 1), as follows (Eq. 2):

(1) $$D\,T-C=\left[\frac{\left(T_{\left(D\,T\right)}-T_{\left(S\,T\right)}\right)}{\frac{\left(T_{\left(S\,T\right)}+\;T_{\left(D\,T\right)}\right)}{2}}\right]\,X\,100$$

*where: DT-C is the cost of the dual task; T_(__*DT*__)_ is the DT duration (in sec), T_(__*ST*__)_ is the ST duration (in sec).*

(2) $$P-i\,n\,d\,e\,x=\left[1-\left[\frac{\left(T_{\left(D\,T\right)}-T_{\left(S\,T\right)}\right)}{\frac{\left(T_{\left(S\,T\right)}+T_{\left(D\,T\right)}\right)}{2}}\right]\right]\times W_{1}+\left(\frac{C\,o\,r\,r\,e\,c\,t}{T\,o\,t\,a\,l}\right)\times W_{2}$$

*where: P-index is the DT performance index from 0 (poor) to 1 (high); T_(__*DT)*_ is the DT duration (in sec), T_(__*ST)*_ is the ST duration (in sec); W_1_ is the weight attributed to time-delta (DT-ST) in the equation (from 0 to 1); Correct is the number of correct cognitive replies; Total is the number of total cognitive replies (correct and wrong); W_2_ is the weight attributed to the accuracy (accuracy = correct/total replies) in the equation (from 0 to 1).*

In this study, the accuracy of cognitive replies was defined by the ratio between the correct number of cognitive replies to the total replies during each DT iTUG phase (Figures 1A,B). We tested the weight of cognitive replies (i.e., correct/total answers) by increasing W_2_ and decreasing W_1_ at 0, 0.1, 0.2, 0.3, 0.4, 0.5, 0.6, 0.7, 0.8, 0.9, and 1, respectively. We have also compared the DT-C estimated by previously published equations (Kelly et al., 2010; Asai et al., 2018; Hunter et al., 2018).

### Statistical Analysis

Data are expressed as mean and standard deviation or median and inter-quartile range, according to their normality profile. Data normality was assessed using the Shapiro-Wilk test. Multiple linear regression and interaction analysis were used to test the ability of the P-index to predict MMSE scores as well as relation of the P-index with other independent clinical and sociodemographic variables. The Receiver Operating Characteristic Curve (ROC curve) was used to determine the weights of the P-index that exhibit the largest area under the curve and ideal cut-off values, in terms of sensibility and specificity, to detect MMSE scores and history of falls. The Bland-Altman analysis was applied to evaluate possible DT-C differences obtained using both time-delta and P-index equations. The within-group and between-group comparisons were assessed using the Friedman and Kruskal-Wallis tests, respectively. The Mann-Whitney U and Wilcoxon tests were also used when indicated. The Spearman’s correlation rank was used to explore possible correlations between the MMSE domains and P-index values. Statistical significance (p) was set at α = 0.05. The Statistical Package for the Social Sciences (SPSS, version 25.0) including the PROCESS macro (version 3.5) and GraphPad Prism (version 9.0) were used to analyze data and generate the graphical representations.

## Results

All the participants completed the assessment. The sample characterization is shown in Table 1.

**TABLE 1:** Sociodemographic and clinical characteristics of the studied sample (*n* = 63).

Variable	Values
Age (mean ± SD)	89.25 ± 4.28
Gender (male/female) (n, %)	7 (11.11)/56 (88.9)
Mean arterial pressure (mean ± SD, mmHg)	93.25 ± 9.37
Schooling (mean ± SD, years)	7.94 ± 4.92
Marital Status (widow/single or married) (n, %)	45 (71.43)/18 (28.57)
Medications in use (mean ± SD, number)	4.77 ± 2.56
Total MMSE score (mean ± SD)	26.47 ± 2.95
Ethnicity (white/brown or black) (n, %)	56 (88.90)/7 (11.11)
Smoking (no/yes) (n, %)	61 (96.82)/2 (3.17)
Drinking (no/yes) (n, %)	52 (82.54)/11 (17.46)
History of falls^*^ (no/yes) (n, %)	32 (50.79)/31 (49.20)

*History of falls: falls occurred in the 6 months prior to the study assessment.

### The Accuracy of Cognitive Replies as a Contributing Factor to Estimate DT Performance (P-Index) in Different iTUG Phases

**FIGURE 2:**
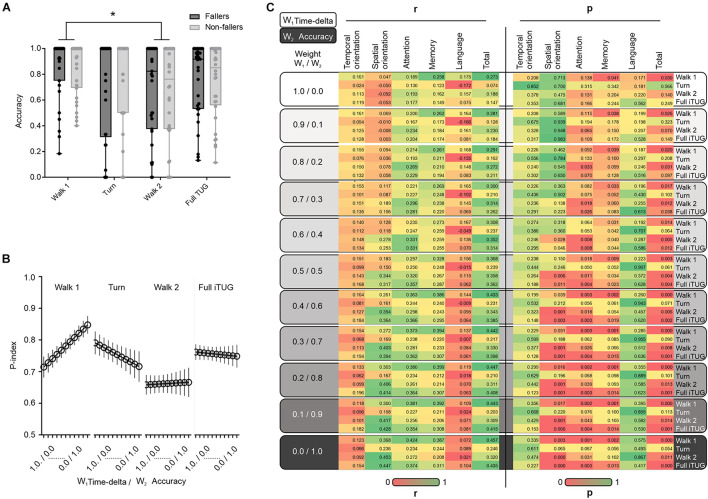
The accuracy of the cognitive replies and DT performance in the iTUG test. **(A)** Between-group (fallers vs. non-fallers) comparison regarding the accuracy of the cognitive replies at different iTUG test phases. Note the difference in the accuracy of cognitive replies between the walk 1 and walk 2 phases. **(B)** The novel P-index is also substantially lower in the walk 2 phase. **(C)** Change in the coefficient of correlation between P-index and MMSE when the weight of the cognitive replies (accuracy; W_2_) is changed by increasing W_2_ and decreasing W_1_ at 0, 0.1, 0.2, 0.3, 0.4, 0.5, 0.6, 0.7, 0.8, 0.9, and 1, respectively. Note the change in the correlation coefficients when the W_2_ weight is increased. In **(A)** Box and whiskers plot showing accuracy of the cognitive replies in the different iTUG test phases (walk 1, turn, walk 2, and full iTUG). The central line is the median, the hinges of the plot are the 25th to 75th percentiles and extremes are min to max values. *p < 0.05.

As shown in Figure 2A, the median accuracy of the cognitive replies differed between the walk 1 and walk 2 iTUG phases. The lower level of accuracy occurred during the walk 2 phase of the iTUG, independently of W_2_. The P-index curves (Figure 2B) revealed that W_2_ produced a notable change in the P-index from the walk 1 and turn phases of the iTUG, while W_2_ effects on walk 2 and Full iTUG phases were less evident (in the walk 2 phase, the P-index value was low regardless of the weight).

The present findings also revealed no major differences between oldest-old with and without a history of falls using both, the time-delta DT-C (Eq. 1) or the novel P-index (Eq. 2) approaches (Asai et al., 2018). We also applied equations described in other studies (Kelly et al., 2010; Hunter et al., 2018), nonetheless, the results also indicated no significant differences between fallers and non-fallers. Both fallers and non-fallers exhibited greater DT-C during the walk 1 and 2 phases (Supplementary Table 1, *p* < 0.05). Although there were no between-group differences in the MMSE scores or the accuracy of the cognitive replies, the latter differed when the iTUG phases were compared (Supplementary Table 2). There were more cognitive errors (lower accuracy of the cognitive replies) during the walk 2 phase when compared to the walk 1 phase (Figure 2A and Supplementary Table 2). Additionally, the ROC curve analysis was used to test whether the time-based DT-C and the P-index equations were able to predict falls in our sample. The findings revealed both equations failed in predicting falls among the studied oldest-old (DT-C equation: *p* ≈ 0.902; P-index: *p* ≈ 0.485; Supplementary Table 3).

### Cognitive Assessment (MMSE) and DT Performance (P-Index)

**FIGURE 3:**
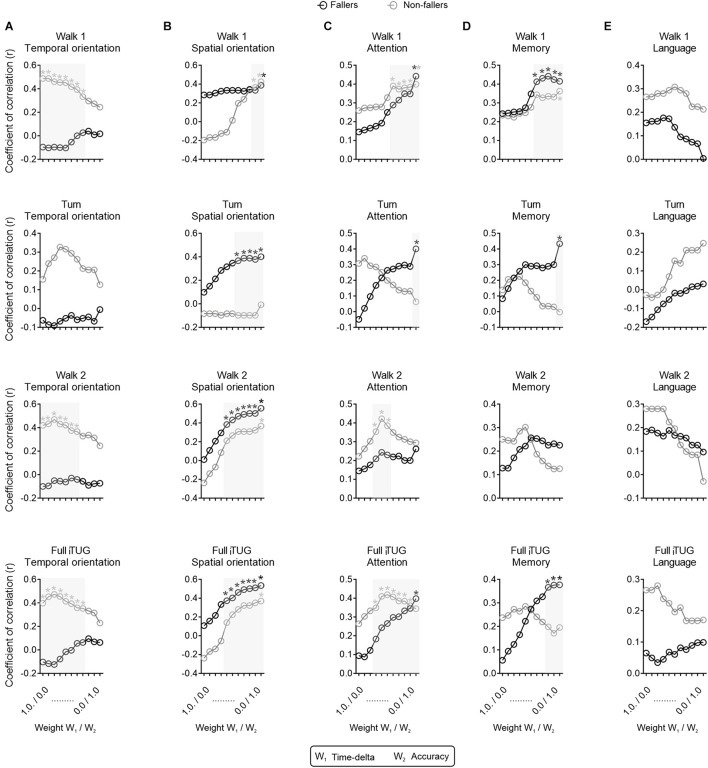
Correlations between the novel P-index and mini-mental state examination (MMSE) domains. Correlation between the novel P-index and the **(A)** temporal orientation, **(B)** spatial orientation, **(C)** attention, **(D)** memory, and **(E)** language domains of the MMSE. *p < 0.05, *r*,Spearman’s rank correlation coefficient.

The exploratory correlation between the P-index values and total scores in the MMSE is shown in Figure 2C. We found the higher the weight attributed to the accuracy of the cognitive replies (W_2_), the better the correlation between the Full iTUG P-index and the MMSE total score. We also noted some MMSE domain-specific relationships with the P-index values across the iTUG phases. The MMSE temporal orientation scores seemed to better correlate with the walk 1 phase, walk 2 phase, and full iTUG assessment, mainly in the non-fallers (Figure 3A). Nonetheless, when adding weight to the W_2_ component of the P-index this effect disappeared. Conversely, greater W_2_ weights led to statistically significant correlations between the P-index and the MMSE in the spatial orientation domain (Figure 3B). Interestingly, only the fallers exhibited P-index values highly correlated with the MMSE spatial orientation domain during the turn phase of the iTUG. The W_2_ weight was also important when correlating the P-index with the attention domain of the MMSE (Figure 3C). The attention domain was more consistently correlated with P-index values in the walk 1 phase and the full iTUG. P-index values were also correlated with the MMSE memory domain, particularly in the fallers during the turn and full iTUG assessment (Figure 3D). Finally, the language domain of the MMSE was not significantly correlated with any P-index values (Figure 3E).

The ROC curve analysis revealed the P-index should use weights of 0.6 for W_1_ (time-delta) and 0.4 for W_2_ (the accuracy of the cognitive replies) to better predict a total MMSE score ≥26. The P-index cut-off, sensibility, and specificity values are shown in Table 2. We used multiple linear regression, stratifying for fallers and non-fallers, to test whether the MMSE domains could predict the P-index. Briefly, the analysis revealed a statistically significant model for fallers [*F*_(3,27)_ = 5.28; *p* = 0.05; *R*^2^ = 0.37] in the full iTUG test. The MMSE domains attention (ß = 0.38; *t* = 2.03; *p* = 0.05) and spatial orientation (ß = 0.37; *t* = 2.03; *p* = 0.05) were predictors of the P-index in the full iTUG. By contrast, temporal orientation (ß = –0.28; *t* = –1.67; *p* = 0.11) was unable to predict the P-index. A statistically significant model [*F*_(2,31)_ = 8.79; *p* = 0.001; *R*^2^ = 0.38] was also found for non-fallers in the full iTUG test. The MMSE domains temporal (ß = 0.56; *t* = 3.79; *p* = 0.001) and spatial orientation (ß = 0.33; *t* = 2.22; *p* = 0.03) predicted the P-index in the full iTUG test for those without a history of falls (Table 3). Notwithstanding, other P-index weights may be used according to the assessment goals. Interaction-based analyses showed age, schooling, and number of medications in use did not influence the total MMSE score in the studied oldest-old (Table 4). The Bland-Altman analysis indicated the traditional time-based DT-C calculation (*W*_2_ = 0) differed from the selected P-index—note the variance in the difference (y) axis. However, we observed that most of the values lie within the 95% limits of agreement, which suggests a good between-method agreement (Supplementary Figure 1).

**TABLE 2:** Receiver Operating Characteristic Curve (ROC curve) to predict a total MMSE score ≥ 26 points^†^.

Measure to predict MMSE ≥ 26	Area	Cut-off value P-index	Sensibility	Specificity	*p*
Full iTUG (P-index^£^)	0.74	0.756	0.72	0.68	**0.001**
Walk 1 (P-index^£^)	0.71	0.823	0.72	0.68	**0.01**
Turn (P-index^£^)	0.66	0.623	0.77	0.63	**0.04**
Walk 2 (P-index^£^)	0.76	0.651	0.67	0.68	**0.001**

*Full iTUG, Instrumented timed up and go including all the test phases; Walk 1, the 3-m forward gait phase of the iTUG; Turn, the 180°-turn phase of the iTUG; Walk 2, the 3-m return phase of the iTUG; P-index, performance index of dual task cost (DT-C) that considers time-delta (W_1_) and the accuracy of cognitive replies (W_2_); W_1_, weight of the time-delta (time difference between DT and ST) in the P-index equation; W_2_, weight of the accuracy of the cognitive replies in the P-index equation; Area, area under the ROC curve; DT, dual task; ST, single task; p, significance level (bold values denote statistical significance).*

*^†^The 26-point total MMSE score is the average of the studied sample.*

*^£^The P-index [0.6 (W_1_)/0.4 (W_2_)] exhibited the largest area under the curve (ROC curve analysis) among all the tested weights (the P-index was tested by increasing W_1_ and decreasing W_2_ at 0, 0.1, 0.2, 0.3, 0.4, 0.5, 0.6, 0.7, 0.8, 0.9, and 1, respectively).*

**TABLE 3:** MMSE variation explained by the P-index using multiple linear regression.

P-index^£^	Group	MMSE domain	R^2^	Adjusted R^2^	Standardized ß	*t*-value	Tolerance	VIF
Full iTUG	Fallers	**Attention**	**0.37**	**0.30**	**0.38**	**2.03**	0.65	1.53
		**Spatial orientation**			**0.37**	**2.03**	0.71	1.41
		Temporal orientation			–0.28	–1.67	0.84	1.19
	Non-fallers	**Spatial orientation**	**0.38**	**0.33**	**0.33**	**2.22**	0.99	1.01
		**Temporal orientation**			**0.56**	**3.79**	0.99	1.01
Walk 1	Fallers	Spatial orientation	**0.20**	**0.14**	**0.47**	**2.35**	0.92	1.09
		Temporal orientation			–0.18	–1.03	0.92	1.09
	Non-fallers	Attention	**0.43**	**0.33**	0.27	1.41	0.58	1.74
		Spatial orientation			0.05	0.28	0.70	1.43
		**Temporal orientation**			**0.38**	**2.12**	0.64	1.49
		Memory			0.09	0.46	0.63	1.59
		**Language**			**0.37**	**2.50**	0.93	1.03
Turn	Fallers	Attention	**0.31**	**0.20**	0.22	1.03	0.62	1.60
		Spatial orientation			0.34	1.70	0.68	1.46
		Temporal orientation			–0.39	–1.76	0.56	1.79
		Memory			0.41	1.91	0.58	1.72
	Non-fallers	**Temporal orientation**	**0.13**	**0.10**	**0.36**	**2.15**	1.00	1.00
Walk 2	Fallers	**Attention**	**0.39**	**0.30**	**0.47**	**2.51**	0.65	1.53
		Spatial orientation			0.30	1.66	0.71	1.41
		Temporal orientation			–0.25	–1.52	0.84	1.92
	Non-fallers	**Spatial orientation**	**0.40**	**0.36**	**0.36**	**2.56**	0.99	1.01
		**Temporal orientation**			**0.56**	**3.91**	0.99	1.01

*Full iTUG, Instrumented timed up and go including all the test phases; Walk 1, the 3-m forward gait phase of the iTUG; Turn, the 180°-turn phase of the iTUG; Walk 2, the 3-m return phase of the iTUG; P-index, performance index of dual task cost (DT-C) that considers time-delta (W_1_) and the accuracy of cognitive replies (W_2_); W_1_, weight of the time-delta (time difference between DT and ST) in the P-index equation; W_2_, weight of the accuracy of the cognitive replies in the P-index equation; MMSE, mini-mental state examination; R^2^, goodness-of-fit measure; Adjusted R^2^, R^2^ adjusted for the number of predictors in the model; standardized ß, standardization of the multiple linear regression coefficient; t-value, calculated t-value in the regression model.*

*Tolerance and VIF (variance inflation factor) are multicollinearity tests (tolerance > 0.1 and VIF < 10 suggests absence of multicollinearity).*

*Temporal orientation, spatial orientation, attention, memory, and language are the MMSE domains. Bold values denote statistical significance or relevant domains in the regression model (ß-based significance).*

*^£^The P-index [0.6 (W_1_)/0.4 (W_2_)] exhibited the best area under the curve (ROC curve analysis) among all the tested weights (the P-index was tested by increasing W_1_ and decreasing W_2_ at 0, 0.1, 0.2, 0.3, 0.4, 0.5, 0.6, 0.7, 0.8, 0.9, and 1, respectively).*

**TABLE 4:** Interaction analyses to predict MMSE scores (total and domains).

Total MMSE	B	p
P-index^£^ ^*^ Age	–0.26	0.55
P-index^£^ ^*^ Schooling	–0.17	0.69
P-index^£^ ^*^ Medications in use	–0.51	0.55

**Temporal orientation**	**B**	**p**

P-index^£^ ^*^ Age	0.04	0.73
P-index^£^ ^*^ Schooling	–0.09	0.36
P-index^£^ ^*^ Medications in use	–0.11	0.06

**Spatial orientation**	**B**	**p**

P-index^£^ ^*^ Age	0.60	0.33
P-index^£^ ^*^ Schooling	–0.43	0.72
P-index^£^ ^*^ Medications in use	–0.27	0.79

**Attention**	**B**	**p**

P-index^£^ ^*^ Age	–0.22	0.31
P-index^£^ ^*^ Schooling	–0.10	0.62
P-index^£^ ^*^ Medications in use	–0.11	0.80

**Memory**	**B**	**p**

P-index^£^ ^*^ Age	–0.04	0.77
P-index^£^ ^*^ Schooling	0.03	0.82
P-index^£^ ^*^ Medications in use	–0.26	0.42

**Language**	**B**	**p**

P-index^£^ ^*^ Age	–0.12	0.10
P-index^£^ ^*^ Schooling	0.09	0.23
P-index^£^ ^*^ Medications in use	–0.12	0.41

*MMSE, mini-mental status examination; P-index, performance index of dual task cost (DT-C) that considers time-delta (W_1_) and the accuracy of cognitive replies (W_2_); W_1_, weight of the time-delta (time difference between DT and ST) in the P-index equation; W_2_, weight of the accuracy of the cognitive replies in the P-index equation; B, Beta regression coefficient; p, significance level. Full iTUG was used to estimate P-index in the interaction analysis.*

*Between-factor interaction.

*^£^P-index [0.6 (W_1_)/0.4 (W_2_)] was selected based on the ROC curve analysis (please see Table 2). Temporal orientation, spatial orientation, attention, memory, and language are MMSE domains.*

## Discussion

This study sought to explore how the accuracy of the cognitive replies in a dual task (DT) influences the task cost (DT-C) by introducing a novel flexible performance index (P-index). This P-index was created by adapting time-delta-based equations commonly used to determine the DT-C. First, this study provides evidence on how the accuracy of cognitive replies changes across the iTUG phases in the oldest-old, regardless of their history of falls in the previous 6 months. Secondly, that weighting the accuracy of cognitive replies in the P-index transferred this effect to the index and afforded a better relation of the novel P-index with neurocognitive scores assessed by the MMSE. The novel P-index enhanced the relationship of the traditional DT-C calculations with cognitive domains related to attention, memory, spatial and temporal orientation.

In terms of the accuracy of the cognitive replies, several factors may explain the difference observed between the walk 1 and walk 2 iTUG phases. The walk 2 phase is preceded by the turn phase (180° turn), which is the most cognitively demanding part of the test (Thigpen et al., 2000; Hollands et al., 2010, 2014). Thus, the accuracy of the cognitive replies in the walk 2 phase is probably influenced by the turn phase, which may explain the difference between the walk 1 and walk 2 phases since they are very similar in terms of biomechanical demand (Courtine and Schieppati, 2003; Al-Yahya et al., 2011; Lowry et al., 2012). This influence is also observed in individuals with Huntington’s disease. The turn phase of the TUG generates a cognitive interference in attention and information processing, which suggests the simultaneous use of motor and cognitive resources increases gait variability during turning movements (Purcell et al., 2020). Similarly, a previous study involving individuals with Parkinson’s disease found an association between processing speed and turning while walking, but no other correlation with cognitive domains (Pal et al., 2016).

To the best of our knowledge, this was the first study to investigate how weighting the accuracy of cognitive replies may impact the DT-C estimation (P-index) in the oldest-old during a mobility task. This effect was not homogeneous across all the test phases. For instance, the greater the weight attributed to the accuracy of the cognitive replies (W_2_) in the walk 1 phase, the more the P-index increased, suggesting good cognitive performance during this walking phase. Indeed, the walk 1 phase, i.e., the initial 3-m-linear walk, does not typically require significant cortical demand from the participants (Malcolm et al., 2015; Wagshul et al., 2019), which allows them to prioritize the cognitive component of the task and, as a result, may increase the success rate in the cognitive replies. Also, the walk 1 phase is performed soon after the participants are told to do their best in saying the weekdays in reverse order, which probably influences the cognitive focus and task prioritization (Verghese et al., 2007; Kelly et al., 2010). By contrast, the turn phase requires a quick 180° change in walking direction, thus increasing the cognitive-motor demand. The act of turning requires higher levels of motor control than linear walking and involves coordinating visual inputs, spatial memory/direction sense, environmental recognition, and biomechanical adjustments (Herman et al., 2011; Shin and Yoo, 2015; Mellone et al., 2016). Consequently, our findings may indicate that the higher cognitive load during the turn movement may have inhibited the focus on the cognitive replies due to the priority given to the motor task (Li et al., 2012; Porciuncula et al., 2016). Interestingly, weighting the accuracy of the cognitive replies in the walk 2 phase did not impact the P-index values, probably because the respondence rate of the cognitive replies was low. The inhibition of the cognitive component in the DT, which typically occurs during the turn phase of the iTUG, may have persisted long enough to hamper the response rate and accuracy during the walk 2 phase (Haid and Federolf, 2019). Further studies may adapt the P-index equation to other types of cognitive tasks with greater ability to deal with low respondence rates, or with greater sensitivity to detect the cognitive load.

Weighting the accuracy of the cognitive replies in the Full iTUG test resulted in a mild effect on the P-index values. In all likelihood, this occurs because the P-index observed in the walk 1 and turn phases are opposing. As a result, the Full iTUG findings are coherent with the patterns of the accuracy-related curves among the overall iTUG test phases. This suggests calculating the P-index without separating the iTUG test phases may lead to the loss of information regarding task-focus predominance during the test.

The proper cognitive-motor DT is expected to overload cognitive domains while performing a movement (Ehsani et al., 2019; Beurskens, 2020). Here, we also examined how weighting the accuracy of the cognitive replies (W_2_) in the P-index correlates with the MMSE scores. When W_2_ is not considered (equal to zero) the correlation between the P-index and MMSE was poor, which suggests the traditional DT-C equations may underestimate the cognitive cost during the DT. The gradual addition of the accuracy of the cognitive replies in the P-index generated dose-response curves indicating the relation of P-index with the MMSE scores. This is an important increment to previous studies highlighting the importance of correcting the performance indexes for cognitive stops and cognitive errors when screening older adults for risk of falls (Tomas-Carus et al., 2019).

The addition of cognitive demand in a DT increases the prioritization levels of cognitive domains such as attention (Maclean et al., 2017). For example, using spatiotemporal gait characteristics, the DT-C displays a positive relationship with a shared attention task (de Bruin and Schmidt, 2010). Our data support the role of attention during the DT, which is seen when weight is added to the W_2_ component of the P-index. Similar results were observed for the fallers in the turn phase of the iTUG. This indicates individuals with higher scores in the attention domain of the MMSE also performed better in the iTUG during DTs. This may be related to the fact individuals with attention deficits and higher risk of falling tend to look away from the target before completing a task (Chapman and Hollands, 2005). Previous studies have highlighted the direct influence of attention on speed and gait adaptation (Maclean et al., 2017; Bayot et al., 2018; Cedervall et al., 2020). Selective attention is highly associated with multitask performance (Harvey, 2019), which agrees with the present findings.

In addition to attention, our study indicated a relationship between the P-index with the memory and spatial orientation domains of the MMSE. These findings were more evident for individuals with a positive history of falls—i.e., a greater relation between P-index and memory and spatial orientation emerged when the weight of the accuracy of the cognitive replies was increased. Gray et al. (2021) found that individuals with poor memory reduce their walking speed by 11% when performing a DT (series of subtractions in a 10-m walking test), while individuals without evident memory deficits by 5%. Similarly, individuals exhibiting symptoms of cognitive decline also perform worse in DTs involving semantic memory, which is responsible for storing, retaining, and evoking long-term information (Theill et al., 2011). Here, our results also indicated that the memory domain (e.g., recording and evocation) exhibited a strong correlation with the P-index, especially in participants with a history of falls. Remarkably, the attention, memory, and spatial orientation domains of the MMSE only influenced the DT-C when the novel P-index was calculated with enough weight to the cognitive replies (W_2_). Overall, this study suggests further trials should consider the full range of weights for both the time-delta (W_1_) and the accuracy of the cognitive replies (W_2_) to ensure a comprehensive DT-C estimation in the oldest-old. Overall, the P-index may be used in association with neuropsychological assessment to enhance the understanding of how cognitive domains influence performance in cognitive-motor DTs. Translating these findings into a clinical setting, we might consider interventions focusing on different cognitive domains to improve ADLs involving DT performance before and after a fall. For instance, interventions focusing on the spatial and temporal orientation domains may be the best strategy for non-fallers, while the attention and spatial orientation cognitive domains could be more appropriate for fallers.

The interaction analysis suggested the P-index [W_1_ (0.6)/W_2_ (0.4)] was not biased toward characteristics such as age, schooling, and medication use. This indicates the P-index could be a good marker to capture the DT-C in walking-based activities. From a clinical perspective, the P-index may help detect cognitive-motor impairments in the oldest-old with different sociodemographic characteristics, thus contributing to improve the assessment of the cognitive-motor functioning—particularly when compared with the traditional DT-C approaches that disregard the accuracy of the cognitive replies. Furthermore, we can speculate that the stability of an individual’s P-index (throughout the full range of W_1_ and W_2_ weights) could indicate their ability to deal with simultaneous cognitive-motor demands. Nonetheless, further trials are needed to test how the P-index could contribute to prevent/diagnose problems in cognitive-motor coupling.

Finally, neither the time- or speed-delta DT-C nor the novel P-index equations revealed between-group differences when the history of falls was considered as a dependent factor. Our findings showed the tested DT-C equations (time-based and P-index) were unable to predict falls in our sample. This is not completely unexpected because some of the oldest-old (people aged ≥ 80 years) change their daily life routine and behavior to minimize exposition to situations involving risk of falls. Thus, we can hypothesize that some non-fallers and fallers exhibit similar cognitive-motor capacity, being only differentiated by their taken-risk behavior profile. However, this secondary analysis should be interpreted with caution since the study was not designed/powered to obtain definitive conclusions on this subject. Also, other biomechanical/clinical factors not usually accounted in the time-based iTUG analyses, such as movement quality, may unveil subtle differences between individuals with and without a history of falls, with respective enhancements in the prediction models (Figueiredo et al., 2020). Moreover, in recent years, there has been considerable debate regarding the DT-C assessment and risk of falls (Bootsma-van der Wiel et al., 2003; Beauchet et al., 2008; Uemura et al., 2011; Tomas-Carus et al., 2019). For example, Bootsma-van der Wiel et al. (2003) assessed 380 oldest-old individuals and reported DT involving vocalizations (animals or professions) was not better at predicting falls compared to a ST, which is consistent with our findings (Bootsma-van der Wiel et al., 2003). Additionally, Beauchet et al. (2008) found no differences between fallers and non-fallers using a backward-counting-based DT (Beauchet et al., 2008). Conversely, Uemura and cols suggested a similar DT was able to differentiate fallers and non-fallers during the steady-state walking (Uemura et al., 2011). Finally, Tomas-Carus et al. (2019) suggested a backward-counting-based DT was able to discriminate fallers and non-fallers after correcting the performance score for cognitive stops and cognitive errors (Tomas-Carus et al., 2019). Overall, further trials are required to determine the optimal tasks and equations to predict falls in the oldest-old.

## Limitations

This study has some limitations. First, the sample was recruited by convenience, thus the participants may not fully represent the oldest-old population. Moreover, the sample characteristics may have influenced the present findings (88.9% of the participants were female, 71.43% single/widow, 88.9% white, 96.2% non-smokers, and 82.54% non-drinkers). Unfortunately, the unbalanced sample profile introduced a statistical impossibility to examine whether these variables could modify the relationship between P-index and MMSE domains, which is a matter for further investigation. Finally, the current findings might differ using other cognitive tasks during the iTUG. Further studies comparing different tasks using the novel P-index curve values may help detect DT-C in the community-dwelling oldest-old.

## Conclusion

A novel P-index to estimate dual task cost (DT-C) was presented, this index uses the weighted components W_1_ (time-delta) and W_2_ (accuracy of the cognitive replies). Dose-response curves indicated that weighting the accuracy of cognitive replies (W_2_) increased the relation of the P-index with the attention, memory, and spatial orientation domains of the MMSE. The novel P-index may assist the mobility assessment by combining cognitive-motor performance during the iTUG test. We identified the turn and walk 2 phases (returning walk) are more cognitively demanding, suggesting the cognitive load at different phases of the test should be considered. The above-mentioned findings may contribute to providing a more accurate assessment of functional mobility in the oldest-old. Importantly, W_1_ and W_2_ may be adapted to other mobility and cognitive tasks, respectively—expanding the applicability of the novel P-index.

## Data Availability Statement

The raw data supporting the conclusions of this article will be made available by the authors, without undue reservation.

## Ethics Statement

The studies involving human participants were reviewed and approved by the Pontifical Catholic University of Rio Grande do SUl Research Ethics Committee (research protocol 2.278.707). The patients/participants provided their written informed consent to participate in this study.

## Author Contributions

FB planned the experiments, performed the data collection, and wrote the first draft of the manuscript. GB elaborated the structure of the model and equation, analyzed the data, draw the figures, wrote the first draft of the manuscript, and reviewed the final version of the manuscript. AF performed the data collection and collaborated with the reasoning of the manuscript. DH analyzed the MMSE domains and collaborate in writing the first draft of the manuscript. AS collaborated in the manuscript reasoning and writing the first draft of the manuscript. RM conceived the study idea, elaborated of the structure of the model and equation, supervised the findings, wrote, and reviewed the second draft of the manuscript. All authors contributed to the article and approved the submitted version.

## Conflict of Interest

The authors declare that the research was conducted in the absence of any commercial or financial relationships that could be construed as a potential conflict of interest.

## Publisher’s Note

All claims expressed in this article are solely those of the authors and do not necessarily represent those of their affiliated organizations, or those of the publisher, the editors and the reviewers. Any product that may be evaluated in this article, or claim that may be made by its manufacturer, is not guaranteed or endorsed by the publisher.

## Supplementary Material

Supplementary Figure 1 Bland-Altman analysis indicated that the novel P-index displays variability in relation to the traditional time-delta index, with good agreement between methods. Bland-Altman plot exhibits 95% limits of agreement between –0.2509 and 0.2614. Note very few dots exceeded the limits of agreement. Bias and standard deviation (SD) of bias were considered low.

Supplementary Table 1 The dual-task cost (DT-C in %) using the traditional time- or speed-delta equations.

Supplementary Table 2 Mini-mental state examination (MMSE) scores and cognitive replies during the dual task (DT).

Supplementary Table 3 Receiver Operating Characteristic Curve (ROC curve) to predict history of falls.
