# Supplementary figures and images for: The Performance Index Identifies Changes Across the Dual Task Timed Up and Go Test Phases and Impacts Task-Cost Estimation in the Oldest-Old
Source: Front Hum Neurosci. 2021 Sep 30;15:720719. doi: 10.3389/fnhum.2021.720719 (PMC8514992; doi:10.3389/fnhum.2021.720719)

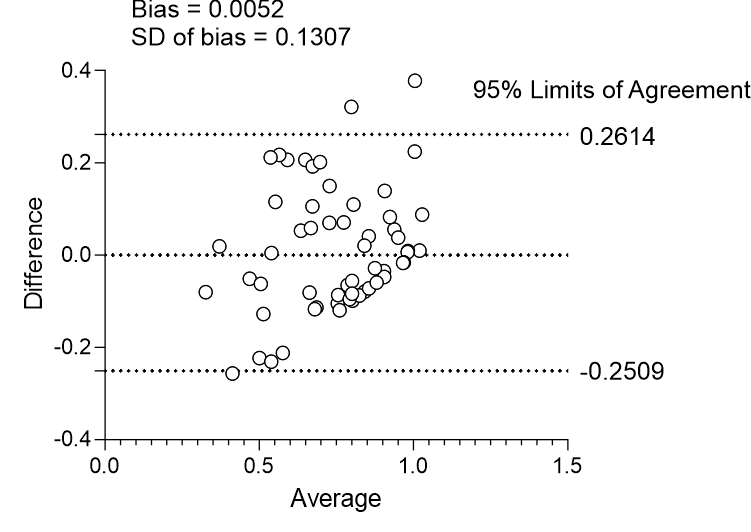

Supplement: Supplementary Figure 1 — Bland-Altman analysis indicated that the novel P-index displays variability in relation to the traditional time-delta index, with good agreement between methods. Bland-Altman plot exhibits 95% limits of agreement between –0.2509 and 0.2614. Note very few dots exceeded the limits of agreement. Bias and standard deviation (SD) of bias were considered low. [file Image_1.TIF]
