# Supplementary material for: The Performance Index Identifies Changes Across the Dual Task Timed Up and Go Test Phases and Impacts Task-Cost Estimation in the Oldest-Old
Source: Front Hum Neurosci. 2021 Sep 30;15:720719. doi: 10.3389/fnhum.2021.720719 (PMC8514992; doi:10.3389/fnhum.2021.720719)
Supplement: Supplementary Table 1 — The dual-task cost (DT-C in %) using the traditional time- or speed-delta equations. [file Data_Sheet_1.docx]

| **Supplementary Table 1.** The dual-task cost (DT-C in %) using the traditional time- or speed-delta equations. | | | | | | | | | | | | |
| --- | --- | --- | --- | --- | --- | --- | --- | --- | --- | --- | --- | --- |
|  |  | **Fallers** | | | | |  | **Non-fallers** | | | | |
| Kelly et al, 2010 |  | Mean |  | SD |  | N |  | Mean |  | SD |  | N |
| Walk 1 |  | 38.487^* **^ |  | 39.043 |  | 31 |  | 40.051^* **^ |  | 42.063 |  | 32 |
| Turn |  | 22.064 |  | 40.138 |  | 31 |  | 35.684 |  | 40.359 |  | 32 |
| Walk 2 |  | 43.398^# ##^ |  | 42.137 |  | 31 |  | 50.854^# ##^ |  | 42.210 |  | 32 |
| Sit-to-stand |  | 14.926 |  | 47.214 |  | 31 |  | 6.978 |  | 18.340 |  | 32 |
| Stand to sit |  | 11.106 |  | 26.702 |  | 31 |  | 21.654 |  | 22.117 |  | 32 |
| Full iTUG |  | 27.147 |  | 29.254 |  | 31 |  | 32.939 |  | 27.484 |  | 32 |
|  |  |  |  |  |  |  |  |  |  |  |  |  |
| Asai et al., 2018 |  |  |  |  |  |  |  |  |  |  |  |  |
| Walk 1 |  | 28.192^*^ |  | 26.234 |  | 31 |  | 28.929^*^ |  | 26.675 |  | 32 |
| Turn |  | 15.059 |  | 28.076 |  | 31 |  | 25.893 |  | 27.465 |  | 32 |
| Walk 2 |  | 31.458^# ##^ |  | 25.456 |  | 31 |  | 36.727^# ##^ |  | 23.897 |  | 32 |
| Sit-to-stand |  | 6.807 |  | 34.722 |  | 31 |  | 5.307 |  | 16.854 |  | 32 |
| Stand to sit |  | 7.587 |  | 24.359 |  | 31 |  | 17.869 |  | 17.469 |  | 32 |
| Full iTUG |  | 21.351 |  | 20.684 |  | 31 |  | 26.145 |  | 18.929 |  | 32 |
|  |  |  |  |  |  |  |  |  |  |  |  |  |
| Hunter et al., 2018 |  |  |  |  |  |  |  |  |  |  |  |  |
| Walk 1 speed |  | 21.498 |  | 19.406 |  | 31 |  | 21.781 |  | 18.677 |  | 32 |
| Walk 2 speed |  | 22.832 |  | 18.918 |  | 31 |  | 27.891^$^ |  | 17.127 |  | 32 |
|  |  |  |  |  |  |  |  |  |  |  |  |  |
| Note: Dual task cost (DT-C) was calculated following previous work by Kelly et al, 2010, Asai et al., 2018, and Hunter et al., 2018. *walk 1 vs. turn; ** walk 1 vs. full iTUG; ^#^turn vs. walk 2; ^##^ walk 2 vs. full iTUG; ^*^walk 1 vs. walk 2. SD: standard deviation. n: sample size. No between-group (fallers vs non-fallers) differences were found (p>0.05). | | | | | | | | | | | | |

| **Supplementary Table 2.** Mini-mental state examination (MMSE) scores and cognitive replies during the dual task (DT). | | | | | | | | | | | | |
| --- | --- | --- | --- | --- | --- | --- | --- | --- | --- | --- | --- | --- |
|  |  | **Fallers** | | | | |  | **Non-fallers** | | | | |
| *MMSE* |  | Mean |  | SD |  | n |  | Mean |  | SD |  | N |
| Temporal orientation |  | 4.548 |  | 0.768 |  | 31 |  | 4.688 |  | 0.644 |  | 32 |
| Spatial orientation |  | 4.839 |  | 0.374 |  | 31 |  | 4.938 |  | 0.246 |  | 32 |
| Attention |  | 3.839 |  | 1.715 |  | 31 |  | 4.000 |  | 1.391 |  | 32 |
| Memory |  | 4.419 |  | 1.361 |  | 31 |  | 4.531 |  | 0.842 |  | 32 |
| Language |  | 8.613 |  | 0.803 |  | 31 |  | 8.938 |  | 0.246 |  | 32 |
| Total |  | 26.258 |  | 3.376 |  | 31 |  | 27.094 |  | 2.turn |  | 32 |
|  |  |  |  |  |  |  |  |  |  |  |  |  |
| *DT performance / cognitive replies* |  |  |  |  |  |  |  |  |  |  |  |  |
| Total vocalizations (number) |  |  |  |  |  |  |  |  |  |  |  |  |
| Sit-to-stand |  | 0.129 |  | 0.318 |  | 31 |  | 0.031 |  | 0.130 |  | 32 |
| Walk 1 |  | 5.129 |  | 2.504 |  | 31 |  | 4.521 |  | 1.394 |  | 32 |
| Turn |  | 1.753 |  | 1.142 |  | 31 |  | 1.427 |  | 0.635 |  | 32 |
| Walk 2 |  | 4.355 |  | 3.839 |  | 31 |  | 4.031 |  | 1.677 |  | 32 |
| Turn and stand to sit |  | 0.247 |  | 0.375 |  | 31 |  | 0.146 |  | 0.338 |  | 32 |
| Full iTUG |  | 11.613 |  | 7.093 |  | 31 |  | 10.156 |  | 3.238 |  | 32 |
|  |  |  |  |  |  |  |  |  |  |  |  |  |
| Correct (number) |  |  |  |  |  |  |  |  |  |  |  |  |
| Sit-to-stand |  | 0.107 |  | 0.303 |  | 31 |  | 0.031 |  | 0.130 |  | 32 |
| Walk 1 |  | 4.107 |  | 1.638 |  | 31 |  | 3.865 |  | 1.676 |  | 32 |
| Turn |  | 1.129 |  | 0.885 |  | 31 |  | 1.083 |  | 0.821 |  | 32 |
| Walk 2 |  | 2.452 |  | 1.368 |  | 31 |  | 2.531 |  | 1.936 |  | 32 |
| Turn and stand to sit |  | 0.204 |  | 0.362 |  | 31 |  | 0.115 |  | 0.312 |  | 32 |
| Full iTUG |  | 8.000 |  | 3.604 |  | 31 |  | 7.625 |  | 4.313 |  | 32 |
|  |  |  |  |  |  |  |  |  |  |  |  |  |
| Errors (number) |  |  |  |  |  |  |  |  |  |  |  |  |
| Sit-to-stand |  | 0.022 |  | 0.120 |  | 31 |  | 0.000 |  | 0.000 |  | 32 |
| Walk 1 |  | 1.022 |  | 2.546 |  | 31 |  | 0.656 |  | 0.874 |  | 32 |
| Turn |  | 0.624 |  | 1.255 |  | 31 |  | 0.344 |  | 0.467 |  | 32 |
| Walk 2 |  | 1.903 |  | 3.863 |  | 31 |  | 1.500 |  | 1.982 |  | 32 |
| Turn and stand to sit |  | 0.043 |  | 0.114 |  | 31 |  | 0.031 |  | 0.130 |  | 32 |
| Full iTUG |  | 3.613 |  | 7.446 |  | 31 |  | 2.531 |  | 3.060 |  | 32 |
|  |  |  |  |  |  |  |  |  |  |  |  |  |
| MMSE: mini-mental state score. Temporal orientation, spatial orientation, attention, memory, and language are MMSE domains. Sit-to-stand, walk 1, turn, walk 2, turn and stand to sit are the iTUG phases. Full iTUG: Instrumented timed up and go including all the test phases. SD: standard deviation. n: sample size. | | | | | | | | | | | | |

**Supplementary Table 3**. Receiver Operating Characteristic Curve (ROC curve) to predict history

| Falls Prediction  Equation 1 (time-delta) | Area | Cut-off value  P-index | Sensibility | Specificity | p |
| --- | --- | --- | --- | --- | --- |
| Full iTUG | 0.49 | NA | NA | NA | 0.93 |
| Walk 1 | 0.50 | NA | NA | NA | 0.95 |
| Turn | 0.49 | NA | NA | NA | 0.90 |
| Walk 2 | 0.52 | NA | NA | NA | 0.83 |
| Falls Prediction  Equation 2 (P-index^£^) | **Area** | **Cut-off value**  **P-index** | **Sensibility** | **Specificity** | **p** |
| Full iTUG | 0.55 | NA | NA | NA | 0.46 |
| Walk 1 | 0.52 | NA | NA | NA | 0.75 |
| Turn | 0.57 | NA | NA | NA | 0.35 |
| Walk 2 | 0.56 | NA | NA | NA | 0.38 |

of falls in the oldest-old.

Note both equations did not present predictive accuracy to detect falls, thus cut-off, sensibility and specificity values are invalid (NA: not applicable). P-index: performance index of dual task cost (DT-C) that considers time-delta (W_1_) and the accuracy of cognitive replies (W_2_). W_1_: weight of the time-delta (time difference between DT and ST) in the P-index equation; W_2_: weight of the accuracy of the cognitive replies in the P-index equation. ^£^The P-index [W_1_ (0.6) / W_2_ (0.4)] was selected to illustrate the analysis (the P-index weights were tested by increasing W_1_ and decreasing W_2_ at 0, 0.1, 0.2, 0.3, 0.4, 0.5, 0.6, 0.7, 0.8, 0.9, and 1, respectively). No statistically significant differences were found.
